# Association of plasma cytokines and antidepressant response following mild-intensity whole-body hyperthermia in major depressive disorder

**DOI:** 10.1038/s41398-023-02402-9

**Published:** 2023-04-21

**Authors:** Michael C. Flux, David G. Smith, John J. B. Allen, Matthias R. Mehl, Andi Medrano, Tommy K. Begay, Brandon H. Middlemist, Brandon M. Marquart, Steven P. Cole, Christina J. Sauder, Christopher A. Lowry, Charles L. Raison

**Affiliations:** 1 Department of Psychology and Neuroscience, University of Colorado Boulder, Boulder, CO 80309 USA; 2 Department of Chemistry and Biochemistry, University of Colorado Boulder, Boulder, CO 80309 USA; 3 Center for Single Cell Biology, Children’s Hospital of Philadelphia, Philadelphia, PA 19107 USA; 4 Department of Psychology, University of Arizona, Tucson, AZ 85721 USA; 5 Department of Psychiatry, University of Arizona, Tucson, AZ 85724 USA; 6 School of Social Work, University of Washington, Seattle, WA 98105 USA; 7 Department of Integrative Physiology, Center for Neuroscience, and Center for Microbial Exploration, University of Colorado Boulder, Boulder, CO 80309 USA; 8 School of Medicine, University of Colorado Anschutz Medical Campus, Aurora, CO 80045 USA; 9 Research Design Associates Inc, Yorktown Heights, New York, NY 10598 USA; 10 Department of Human Development and Family Studies, School of Human Ecology, University of Wisconsin–Madison, Madison, WI 53703 USA; 11 Department of Physical Medicine and Rehabilitation and Center for Neuroscience, University of Colorado Anschutz Medical Campus, Aurora, CO 80045 USA; 12 Veterans Health Administration, Rocky Mountain Mental Illness Research Education and Clinical Center (MIRECC), Rocky Mountain Regional Veterans Affairs Medical Center (RMRVAMC), Aurora, CO 80045 USA; 13 Military and Veteran Microbiome: Consortium for Research and Education (MVM-CoRE), Aurora, CO 80045 USA; 14 Department of Psychiatry, School of Medicine and Public Health, University of Wisconsin–Madison, Madison, WI 53706 USA

**Keywords:** Depression, Physiology, Human behaviour, Predictive markers

## Abstract

Whole-body hyperthermia (WBH) shows promise for the treatment of major depressive disorder (MDD). Because MDD is associated with increased inflammation, and anti-inflammatory agents show some promise as antidepressants, the current study sought to identify the acute and longer-term immune effects of WBH in participants with MDD and to explore whether these effects associate with the procedure’s antidepressant properties. Thirty participants who met DSM-IV-TR criteria for MDD were randomized to receive a single session of WBH (*n* = 16) or sham treatment (*n* = 14). Hamilton Depression Rating Scale (HDRS) scores were assessed at baseline and 1, 2, 4, and 6 weeks post-treatment (WBH vs. sham), and plasma cytokine concentrations were assessed at baseline, immediately post-treatment, and 1 and 4 weeks post-treatment. As previously reported, WBH produced a rapid and sustained antidepressant effect. When compared to sham, WBH increased plasma interleukin (IL)-6 immediately post-treatment (time by treatment: *χ*^2^_(3, *N*=108)_ = 47.33, *p* < 0.001), while having no effect on other cytokines acutely and no impact on IL-6, or any other cytokine, at 1 or 4 weeks post treatment. In the study sample as a whole, increased IL-6 post-treatment was associated with reduced HDRS depression scores over the 6 weeks of follow-up (*F*_(1, 102.3)_ = 6.74, *p* = 0.01). These results suggest a hitherto unrecognized relationship between hyperthermia, the immune system, and depression, and may point to WBH as a novel modality for exploring behavioral effects of IL-6 when the cytokine is activated in isolation from the inflammatory mediators with which it frequently travels.

## Introduction

Whether in saunas, sweat lodges, or baths, heat has been used since time immemorial for emotional and physical healing. Recent scientific studies support the wisdom of these ancient practices. For example, repeated sauna use has been associated with long-term protection from cardiovascular and all-cause mortality, as well as dementia [1]. In regards to mental health, sauna use associates with a reduced risk of new-onset psychosis [2], hot baths improve symptoms in autistic individuals [3], and hyperthermic baths and whole-body hyperthermia (WBH) have been repeatedly reported to improve symptoms of major depressive disorder (MDD) [4–6]. These findings are consistent with other studies demonstrating that physical warmth promotes feelings of social connection and safety from threat, both of which are protective factors against the development of MDD [7, 8].

These findings highlight the potential value of identifying mechanisms that account for these hyperthermia-induced mental health benefits. Multiple lines of evidence point to the immune system as an attractive candidate in this regard [9]. First, when activated the immune system induces hyperthermia, and this process has been associated with short-term symptomatic improvement in patients with MDD and autism [10, 11]. Second, hyperthermia stimulates immune functioning, which provides a likely partial explanation for the evolution of fever as an anti-pathogen defense [12]. Third, MDD has been repeatedly associated with chronic increases in inflammatory biomarkers (especially tumor necrosis factor [TNF], interleukin-6 [IL-6] and IL-1-beta) [13, 14], and anti-inflammatory agents have been reported to reduce depressive symptoms in patients with increased inflammation [9].

The effects of WBH on the immune system when applied clinically appear to vary by the intensity of heat delivered. High-intensity WBH (e.g., to core body temperature between 41 °C and 42 °C), such as is typically used as an adjunct treatment in cancer, reliably induces a time-limited increase in plasma concentrations of most proinflammatory cytokines [15], whereas the effects of sauna bathing or mild to moderate-intensity WBH (e.g., to core temperature up to 39 °C) have been reported to be primarily anti-inflammatory [16, 17]. However, studies to date of WBH have been conducted in medically ill populations, leaving open the question of whether mild to moderate-intensity WBH would produce a similar response in medically-healthy individuals with MDD.

To explore this question, and to evaluate whether acute and long-term immune responses to WBH are associated with the procedure’s antidepressant effects, we utilized data from a double blind, randomized, sham-controlled trial to examine the impact of a single treatment with mild-intensity WBH on levels of circulating pro- and anti-inflammatory cytokines in participants with MDD immediately after, and at 1 and 4 weeks after, the intervention. Given that participants who received WBH in this study showed a rapid and sustained antidepressant response when compared to sham treatment [5], we hypothesized that the antidepressant effects of WBH would be associated with both immediate and longer-term anti-inflammatory effects of the intervention, measured primarily as reductions in plasma concentrations of IL-6, TNF and IL-1-beta, the inflammatory cytokines most frequently associated with MDD [13, 14].

## Materials and methods

Possible risks and benefits of study participation were discussed with, and signed informed consent was obtained from, all participants after a full description of study procedures and prior to conducting any study procedures. The study was approved by the University of Arizona Institutional Review Board (IRB), was considered exempt by the IRB of the University of Colorado Boulder and was registered on ClinicalTrials.gov as NCT01625546. Details of the study design, primary behavioral outcomes, and occurrence of adverse events have been previously reported [5].

### Participants

**Fig. 1:**
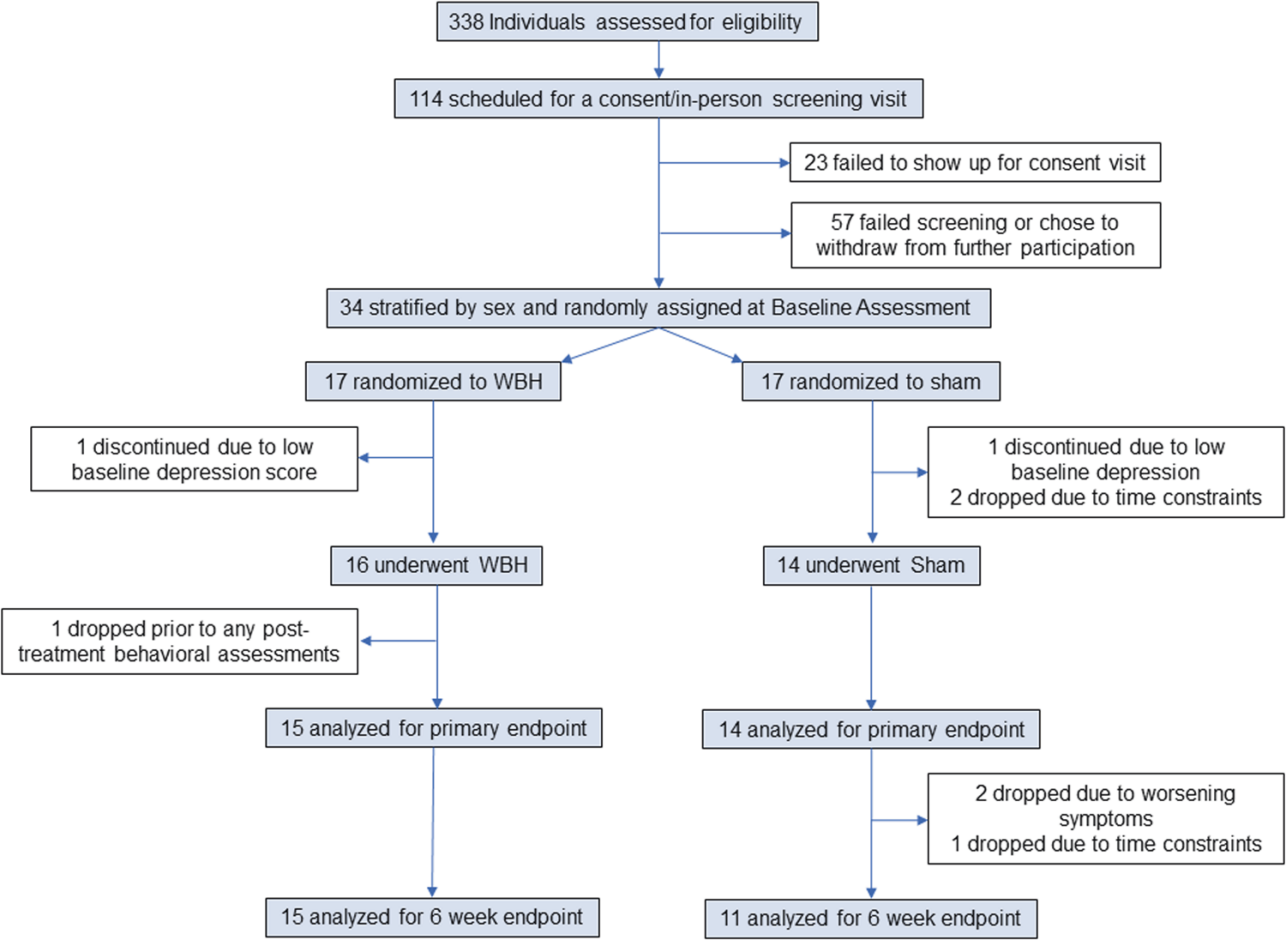
CONSORT trial flow diagram. WBH whole-body hyperthermia.

This study enrolled participants at the Banner University Medical Center in Tucson, Arizona, between February 2013 and May 2015. Participants were recruited via print, radio, posted fliers, email listserv, social media, and television advertising. Eligible participants were males and females, aged 18 to 65 years, who were medically healthy, met Diagnostic and Statistical Manual of Mental Disorders, Fourth Edition, Text Revision (DSM-IV-TR) criteria for MDD lasting at least 4 weeks prior to signing the consent form, and had a baseline 17-item Hamilton Depression Rating Scale (HDRS) score ≥16. All participants were free of psychotropic medications at the time of signing consent and remained off these medications during study participation. A full list of inclusion and exclusion criteria, as well as relevant demographics for the study population, has been provided previously [5]. Fig. 1 provides a CONSORT diagram of participant flow through the study.

### Randomization and blinding

A computer-generated randomization list provided to the study by the Arizona Statistics Consulting Laboratory was used to randomize participants with a one-to-one allocation in blocks of six to a single treatment of WBH or a sham WBH condition. This list was kept by a Psychiatry Department administrator who had no contact with study participants and no other role in the study. Participants remained blinded to their randomization status until completion of the last study assessment at post-treatment week 6.

### Study design

Eligible participants were scheduled to receive an intervention within 25 days of completing screening. Between screening and baseline assessment, participants completed the Inventory of Depressive Symptomatology-Self Report (IDS-SR) at home an average [standard deviation, (SD)] of 8.28 [4.17] days after screening. Participants showing a ≥30% reduction from their IDS-SR score at screening were considered likely responders to non-specific aspects of study enrollment or as demonstrating regression to the mean/spontaneous recovery and were discontinued from the study. On the intervention day, participants arrived at the medical center at 8 AM and completed a baseline assessment that included the HDRS and a blood draw. The study intervention commenced between noon and 1 PM. On completion of the intervention, participants rested for 1 h and were released to home. Follow-up HDRS assessments were conducted at post-intervention weeks 1, 2, 4, and 6. Blood was withdrawn at 8:30 AM prior to receiving a study intervention, immediately (within 30 min) upon completion of the study intervention, and at 1- and 4-weeks post-intervention. With the exception of the immediate post-treatment blood draw, blood was collected between 8:30 AM and 9:30 AM to control for potential circadian variation in plasma cytokine concentrations.

### Study interventions

For both WBH and the sham condition, the current study used a Heckel HT3000 WBH system (Heckel Medizintechnik GmbH and Hydrosun Medizintechnik GmbH). Participants randomized to active WBH received heating at the level of the chest by infrared lights and at the level of the lower extremities by infrared heating coils until their core body temperature reached 38.5 °C, which is the upper limit of core body temperature for mild-intensity WBH [18]. When core body temperature reached 38.5 °C, the infrared lights and heating coils were turned off, and participants remained recumbent in the Heckel device and entered a 60-min cool-down phase. Heart rate and core and skin temperatures were monitored continuously throughout the procedure (see below for detailed procedures).

All procedures for the sham condition were identical to WBH, except that the primary infrared lights were not turned on, and orange-colored non-heating lights were placed at the level of the chest to produce a similar color, but no heat. To increase believability, mild heat was provided within the Heckel device by activating heating coils situated above the participants’ lower extremities at the same setting used for active WBH. Finally, a false fan was used to produce noise, as when the device was fully operational. The duration of exposure to the sham WBH condition was matched to the duration of time that the prior participant of the same sex undergoing actual WBH spent in the active heating phase.

### Outcome measures

#### Behavioral measures

The study was designed to evaluate potential associations between WBH-induced changes in depressive symptoms, core body temperature, and immune markers. For depressive symptom assessment, the current study used the 17-item HDRS [19], which was the primary outcome measure of the parent study [5]. The 17-item HDRS was administered at pretreatment baseline and 1, 2, 4, and 6 weeks post-WBH versus sham WBH. Trained raters blind to group assignment with good inter-rater reliability performed all HDRS assessments [5].

#### Cutaneous and core body temperature

A self-inserted rectal probe (Mindray North America, Mahwah, NJ, USA) remained in place during the WBH session in order to monitor core body temperature during the intervention.

#### Blood collection and preparation of plasma

Cytokine concentrations were measured in plasma via blood samples collected by venipuncture using 10-mL lavender-top EDTA vacutainer tubes (Cat. No. 366643; Becton, Dickinson, and Company (BD), Franklin Lakes, NJ, USA). Samples were centrifuged for 10 min at 1000 × *g* at 4 °C. Plasma was collected, aliquoted, and stored at ‒80 °C until shipment on dry ice to the University of Colorado Boulder; samples were again stored at ‒80 °C until assay.

#### Cytokine measurements

To evaluate the effect of WBH on peripheral inflammation, we used a high-sensitivity 9-plex multiplexed ELISA (Cat. No. 85-0217, Aushon Biosciences, Billerica, MA, USA; now provided as a 10-plex, Cat. No. 85-0002 by Quanterix, Lexington, MA, USA). Use of the multiplex allowed for an examination of a range of circulating cytokines, including interferon (IFN)-gamma, interleukin (IL) 1-alpha, IL-1-beta, IL-4, IL-6, IL-8, IL-10, IL-12p70 (heterodimer), and tumor necrosis factor (TNF; active trimer). Assays were conducted using duplicate samples according to manufacturer’s instructions, and concentrations were measured using the Aushon Signature Plus^TM^ Imaging and Analysis System (Aushon Biosciences). All samples were run simultaneously in duplicate in a single assay, across three 96-well plates (using a separate standard curve for each plate). To minimize variability across time for samples from individuals, all samples from each participant were run on a single plate; in addition, samples from participants in sham and WBH conditions were balanced across the three plates. Intra-assay coefficients of variation, limits of detection, and lower and upper limits of quantification for each cytokine are provided in Supplemental Information.

### Statistical analysis

Participants from the parent study [5] were included in the current study if they completed at least two blood draws, one of which needed to be at baseline and one immediately following the WBH or sham treatment. Thirty-four participants were randomly assigned to either WBH or sham treatment. Four participants were randomized to treatment but were discontinued due to low baseline depression scores or withdrew from the study due to time constraints, leaving 30 viable participants. Frequency distributions, means, and SDs were calculated for the behavioral and immune measures for all endpoints. Distributions were examined for extreme outliers and for significant deviations from normality. Outliers were assessed using the generalized extreme studentized deviant test [20]. All immune biomarker concentration distributions significantly deviated from normality, thus biomarker concentrations were subjected to a natural log transformation, bringing distributions closer to normality. Log transformed biomarker concentrations were used in all models. Analyses using core body temperature used maximum core temperature reached during the course of WBH or sham treatment. Linear mixed models (LMMs) were used to determine the effect of WBH versus sham treatment on plasma biomarker concentrations, as well as on HDRS scores. A Bonferroni correction was applied to address multiplicity, given assessment of the three primary inflammatory cytokines of interest in the cytokine panel (e.g., IL-1-beta, IL-6, TNF). LMMs were used to evaluate the association of multiplex-assessed plasma cytokine concentrations with longer-term effects of WBH vs. sham on HDRS score. These models explored whether changes in HDRS score over time and by treatment condition were moderated by immune biomarker concentrations immediately post-intervention, while controlling for baseline biomarker concentration values. Chi square tests were implemented for complex LMM comparisons, such as when exploring time effects across multiple time points. All tests were two-tailed, with significance set at *p* < 0.05. Analyses were conducted with R version 3.6.1 for Mac OS X. LMMs were constructed using the “lme4” package in R. Code availability can be accessed by contacting corresponding author.

## Results

**Fig. 2:**
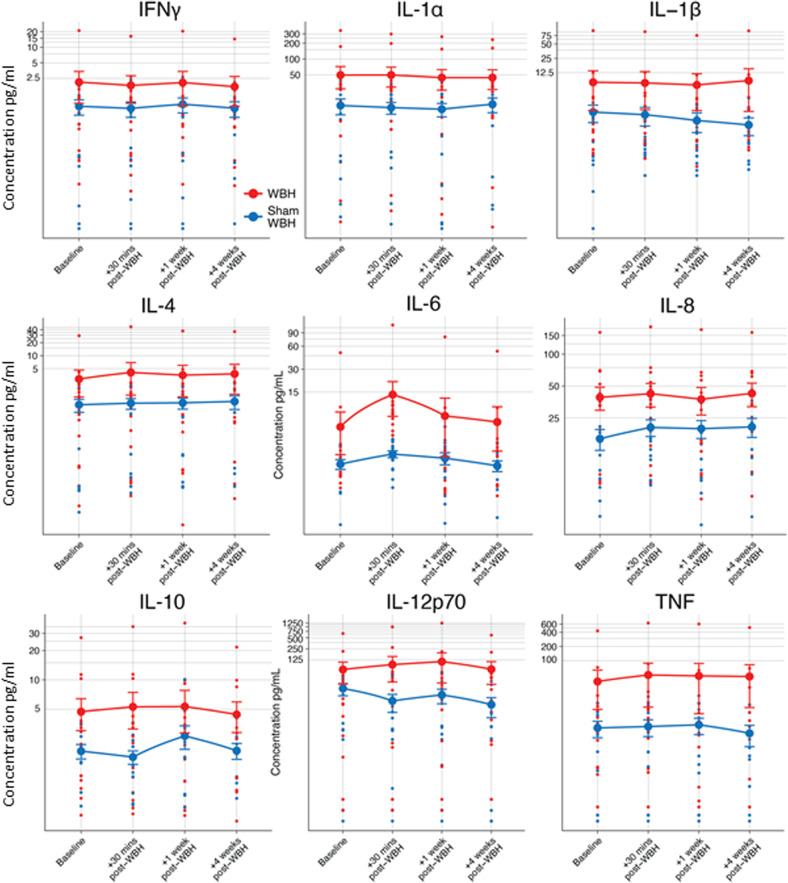
Changes in plasma cytokine concentrations as measured by multiplex enzyme-linked immunosorbent assay (ELISA) in response to whole-body hyperthermia (WBH; red) and sham WBH (blue). Data are presented as scatter plots showing individual values (smaller points) and as means (larger points connected by a line) ± standard deviation. The *y* axis is displayed using a log scale to better display the range of concentration values measured while including participants with elevated inflammation and to reflect that log transformed data were primarily used in all statistical modeling. IFNγ interferon gamma, IL interleukin, TNF tumor necrosis factor, WBH whole-body hyperthermia.

**Fig. 3:**
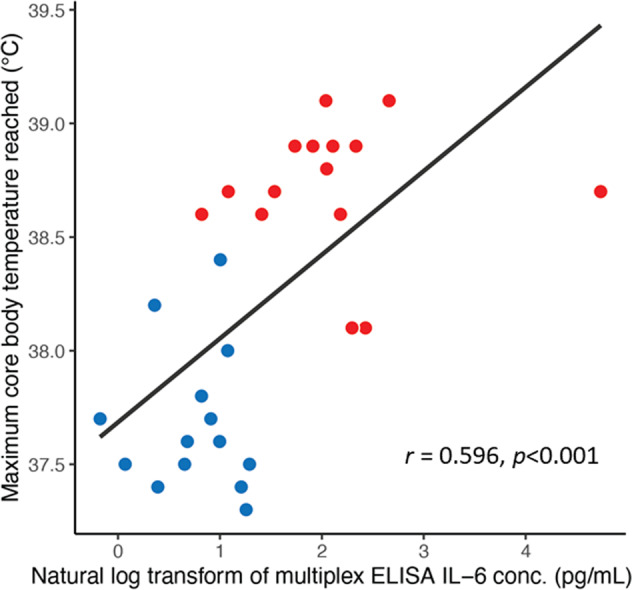
Correlations between natural log transformed plasma concentrations of interleukin (IL)-6 and maximum core body temperature reached during the experiment. Correlation strength (*r*) and significance (*p*) accompany the regression line. Red points refer to data measured from participants randomized to the whole-body hyperthermia (WBH) condition, and blue points refer to data measured from participants randomized to the sham WBH condition. C Celsius, ELISA enzyme-linked immunosorbent assay, pg picogram, mL milliliter, WBH whole-body hyperthermia.

Baseline demographic and behavioral data, as well as mean baseline and intervention-induced core body temperatures are presented in Table 1. As previously reported [5], WBH produced a rapid and sustained reduction of HDRS score when compared to sham treatment in the subset of participants with available cytokine/body temperature data (WBH: *n* = 15; sham: *n* = 14). With the exception of IL-6, WBH had no effect on any log transformed cytokine concentrations, either acutely (i.e., immediately post-treatment) or at weeks 1 and 4 post-treatment (*p*, n.s.; Table S1). Analysis of log transformed IL-6 concentrations using LMM revealed a significant treatment x time interaction (*F*_(1,76.2)_ = 165, *p* < 0001), such that IL-6 increased significantly immediately following WBH, while showing no difference from sham at post-treatment weeks 1 and 4 (Fig. 2; Table S2). Likewise, analysis of log transformed IL-6 concentrations using chi squared tests across all time points showed an effect of treatment x time (χ^2^_(3,*N*=112)_ = 25.21, Bonferroni-corrected *p* < 0.001) (Fig. 2). While WBH increased core body temperature more than the sham condition (*p* < 0.001), it should be noted that the provision of mild heat in the sham condition nonetheless produced an average [SD] increase in core body temperature of 0.78 °C [0.36] in those randomized to sham. As shown in Fig. 3, increasing maximal core body temperature achieved during both the sham and WBH conditions was strongly associated with post-intervention log transformed IL-6 plasma concentrations (*r*(27) = 0.596, *p* < 0.001).

**Table 1:** Demographics, baseline clinical characteristics, and baseline and WBH-induced changes in core body temperature.

	Sham (*n* = 14)	WBH (*n* = 15)
General demographics		
Women *n* (%)	8 (57.1)	11 (73.3)
Mean age in years (±SD)	41.1 (12.5)	38.5 (15.2)
Age range in years	24–61	18–65
White, non-Hispanic *n* (%)	8 (57.1)	9 (60.0)
Clinical characteristics, means (±SD)		
HDRS Baseline	22.6 (3.7)	21.8 (4.5)
Length of depressive episode (months)	9.9 (13.0)	7.2 (11.0)
Temperature characteristics, means (±SD)		
Mean baseline core body temperature	37.0 (0.5)	37.0 (0.3)
Maximum core body temperature	37.7 (0.3)	38.7 (0.3)
Mean core body temperature change	0.7 (0.4)	1.7 (0.4)

*SD* standard deviation, *WBH* whole-body hyperthermia, *HDRS* 17-item Hamilton Depression Rating Scale.

**Fig. 4:**
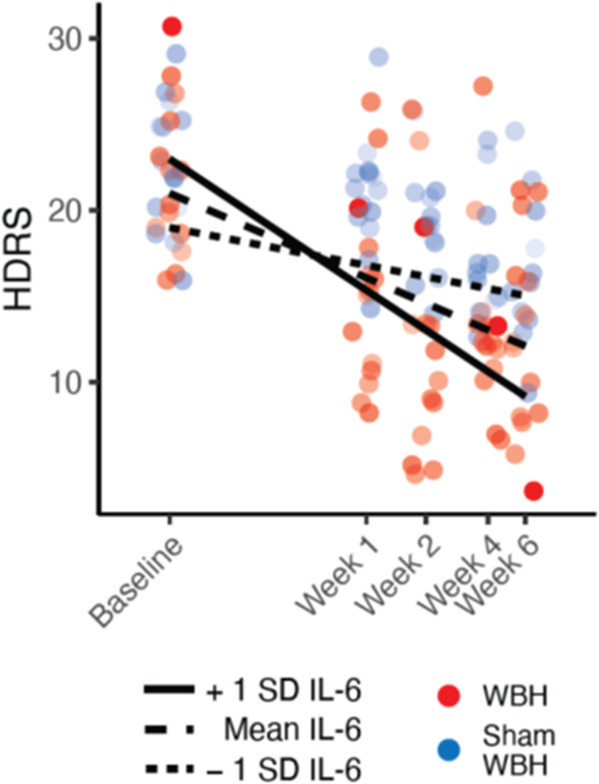
Changes in the Hamilton Depression Rating Scale (HDRS) over time as moderated by varying concentration levels of interleukin (IL)-6 assessed immediately post-whole-body hyperthermia (WBH). Time along the *x*-axis is labeled accurately but displayed on a natural log scale to reflect how the data were analyzed. Data points have been offset slightly (“jittered”) from their original positions to reveal overlapping data points. Red points indicate data measured from participants randomized to the WBH condition, and blue points indicate data measured from participants randomized to the sham WBH condition. Point opacity is based on IL-6 concentrations, with highest concentrations being the most opaque. The solid line represents the relationship between HDRS and time when cytokine concentrations are one standard deviation (SD) above the mean, the long-dashed line represents the same relationship when cytokine concentrations are at their mean value, and the short-dashed line represents that relationship when cytokine concentrations are 1 SD below the mean.

**Fig. 5:**
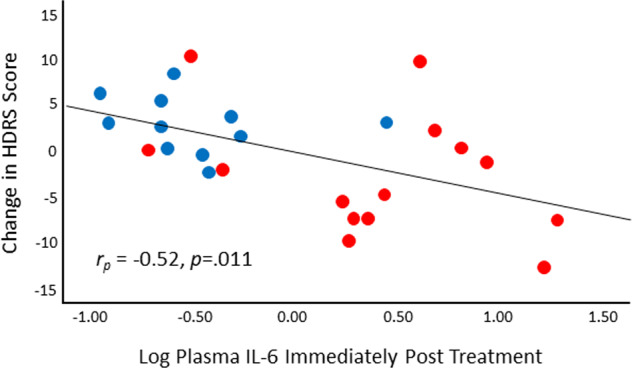
Partial regression plot illustrating the relationship between residuals of the natural log transformed plasma concentrations of interleukin (IL)-6 and residualized change in Hamilton Depression Rating Scale (HDRS) score. The Pearson correlation between the two sets of residuals is equal to the partial correlation between HDRS scores at week 2 post-WBH/sham treatment and the natural log transformed concentration of plasma IL-6 assessed immediately post-WBH/sham treatment (controlling for baseline HDRS scores and baseline natural log transformed concentration of plasma IL-6, respectively). HDRS Hamilton Depression Rating Scale, IL interleukin, WBH whole-body hyperthermia.

To examine whether the longer-term antidepressant effect of WBH was moderated by this cytokine response, the model detailed in Table S3 was used. Using this model structure, acute IL-6 elevations moderated HDRS scores over the 6-week follow-up period in the study sample as a whole (*F*_(1,102.3)_ = 6.74, *p* = 0.011), such that larger increases in IL-6 immediately post-treatment (adjusting for pre-treatment values) were associated with a steeper reduction in depressive symptoms over the follow up period (Fig. 4). However, no group x time x acute cytokine concentration interaction was observed using the model detailed in Table S3 Correlations between acute post-intervention log transformed IL-6 concentrations with HDRS scores 1, 2, 4, and 6 weeks post-WBH identified the strongest correlation (when adjusting for baseline) to be with the 2-week post-treatment time point, as illustrated in Fig. 5 (*r*_*p*_ = –0.522, *p* = 0.011).

## Discussion

This examination of cytokine responses to a single session of mild-intensity WBH versus sham treatment in participants with MDD provided disconfirmatory evidence for the a priori hypothesis that WBH would produce an antidepressant effect in association with reductions in peripheral inflammation. Indeed, within the constraints imposed on our ability to detect smaller effect sizes because of the modest study sample, WBH had no long-term effect on any of the assayed immune biomarkers. On the other hand, WBH produced a large effect size immediate increase in IL-6 that resolved completely within a week of treatment. This acute IL-6 response was shown to moderate the effect of treatment, such that, in the study sample as a whole, larger immediate IL-6 responses to treatment (WBH and sham groups combined) were associated with reduced HDRS depression scores over the 6-week follow-up period, with this effect being numerically maximal at post-treatment week 2 when examined by simple correlations. We suspect that the failure to observe a group x time x post-intervention IL-6 interaction for this effect results from the fact that our sham condition might be better characterized as low-dose WBH. The provision of heat in the sham condition—while useful for blinding—significantly raised core body temperature in many of the subjects, and thereby blurred between-group differences in acute IL-6 response. In fact, two participants in the sham group reached a higher maximum core body temperature than the core body temperature reached by WBH participants who had the smallest increase in core body temperature with treatment.

The finding that increased circulating levels of IL-6 were associated with reduced depression seems counter-intuitive given multiple lines of evidence implicating IL-6 in the pathogenesis of MDD. Indeed, increased baseline circulating IL-6 is among the most replicable immune findings in individuals with MDD who are otherwise healthy [21]. Studies report increased baseline IL-6 in the cerebrospinal fluid of individuals with MDD as well [22], and peripheral levels of IL-6 are inversely correlated with thickness of prefrontal cortex in individuals with MDD [23]. Central nervous system administration of IL-6 in mice produces depressive like-behavior [24], and humans challenged with endotoxin show a significant increase in IL-6 that correlates with the severity of depressive response to the immune stimulus [25]. Finally, IL-6 neutralizing antibodies have been reported to demonstrate an antidepressant effect in patients with elevated inflammation in the context of an autoimmune condition [26].

These findings must be balanced against an intriguing set of contradictory observations, consistent with the fact that IL-6 is a pleiotropic cytokine with anti-inflammatory and neurotrophic effects, in addition to its proinflammatory effects [27]. The cytokine response to WBH closely parallels that seen in response to acute exercise, which also produces a large and time-limited increase in IL-6 with either no, or a modest, impact on TNF, depending on the study [28]. Like WBH, exercise induces acute hyperthermia and produces an antidepressant effect. Although to our knowledge no data link exercise-induced increases in IL-6 to either acute or longer-term effects on mood, one study found that exercise withdrawal induced depressive symptoms that were associated with reduced levels of circulating IL-6 compared to participants who continued to exercise [29].

Importantly, exercise is not the only antidepressant or mood elevating modality reported to induce acute increases in IL-6. Electroconvulsive therapy (ECT), bright light exposure, sleep deprivation, fasting, and ketamine have also been reported to have this effect [30–33]. Fasting, which has been repeatedly shown to promote positive mood [34], induces IL-6 while simultaneously suppressing IL-1-beta activation [35, 36]. Interestingly, ketamine has also been shown to reduce inflammatory activity (i.e., Th17 cells) while simultaneously inducing IL-6 expression [37].

As with ketamine and fasting, it is possible that the association between increased IL-6 and reduced depression in response to WBH might reflect the ability of IL-6 to induce anti-inflammatory cytokines with neurotrophic properties, in particular IL-4, which promotes stress resilience, neurogenesis, and protection against the damaging effects of central nervous system inflammation [38–44]. In this regard, it is interesting to note that although we did not observe an acute increase in plasma IL-4 post-WBH when compared to sham, exploratory linear mixed modeling indicated that, on a within-subjects basis, IL-4 immediately post-treatment was one of several cytokines associated with reduced HDRS depression scores across the 6-week follow-up period (*F*_(1,97.9)_ = 5.61, *p* = 0.020).

Nonetheless, both the sources and therapeutic relevance of IL-6 induction in response to WBH and other antidepressant modalities are poorly understood. Nor is it known whether increases in IL-6 reflect enhanced biological signaling or are a compensatory response to inhibition of IL-6-inducible downstream pathways, such as STAT3 [45]. In the context of these unknowns, mild-intensity WBH may hold promise not just as an antidepressant modality, but also as a probe for understanding the specific effects of IL-6 when the cytokine is divorced from the other immune molecules with which it typically travels. Moreover, should future studies confirm that WBH improves depressive symptoms at least in part via the acute activation of IL-6 signaling, this might encourage a search for other modalities—both behavioral and pharmacologic—that confer a longer-term antidepressant benefit via the same mechanism. Similarly, it will be important to look at the role of acute IL-6 signaling in other hyperthermic interventions with an antidepressant signature, including hot baths and hot yoga.

The current study has a number of strengths [e.g., believability of the sham condition, i.e., 71% of sham-treated patients believed they received active WBH [5], repeated assessment of both immune and behavioral variables]; however, several limitations warrant discussion. Primary among these is the modest sample size, which constrains our ability to detect any but large effect size associations between immune and behavioral responses to WBH; furthermore, the modest sample size may increase the risk for false positive results, although the magnitude of the effect of WBH on IL-6 reduces this risk. Nonetheless, an important next step will be to examine whether current results are confirmed in larger sample sizes. The modest sample size likely also led to randomization failing to create groups with equivalent plasma cytokine concentrations at baseline (with WBH being generally higher). However, the potential impact of these between group differences was mitigated by adjusting for baseline cytokine levels in our statistical analyses. In addition, both the WBH and sham WBH groups received enough heating to raise core body temperature, likely reducing our ability to demonstrate that the association of the acute IL-6 response with reductions in depression was specific to participants randomized to WBH. To address this issue, it will be important to conduct follow-up studies that add a sham comparator without heat, which, while likely reducing credibility and blinding, will better characterize the full acute effect of WBH on circulating levels of IL-6.

An additional limitation is the fact that acute post-treatment immune measures were only assessed once, immediately after the WBH session, and not assessed again until a week later. Because of this, it cannot be determined whether the effect of WBH on circulating cytokines was maximal at the time point of our assessment, raising the possibility that other cytokines may have shown an association with WBH at later time points in the acute response. Subsequent studies that more completely characterize the time course of the acute cytokine response (i.e., within the first 24 h) to WBH may therefore uncover additional effects and/or relationships not apparent from our one assessment immediately post-WBH. Finally, a larger sample size might have allowed the detection of smaller effects of WBH on longer-term plasma cytokine concentrations and/or smaller associations between such effects and changes in depression. In future studies, it will also be important to explore other longer-term mechanisms that may have been activated by the acute IL-6 stimulus, including immune effects in targeted tissues (i.e., brain) and/or non-immune pathways.

In conclusion, results from this study suggest a hitherto unrecognized relationship between hyperthermia, the immune system, and depression, and may point to WBH as a potential modality for exploring behavioral effects of IL-6 in isolation from the inflammatory mediators (e.g., TNF, IL-1-beta) that activate the cytokine in the context of acute infection or in response to the triggers for chronic inflammation (e.g., obesity, sedentary lifestyle) that are all too common in the modern world. Future research should seek to replicate these findings in a larger participant sample and more thoroughly characterize the complete time course of the acute cytokine response to WBH. Finally, the availability of commercially available IL-6 antagonists, raises the interesting possibility that blocking the acute IL-6 response might attenuate longer-term antidepressant effects of the procedure.

## Supplementary information

Supplementary Information
