# Supplementary material for: Association of plasma cytokines and antidepressant response following mild-intensity whole-body hyperthermia in major depressive disorder
Source: Transl Psychiatry. 2023 Apr 21;13:132. doi: 10.1038/s41398-023-02402-9 (PMC10121589; doi:10.1038/s41398-023-02402-9)
Supplement: Supplementary file 1 — Supplementary Information [file 41398_2023_2402_MOESM1_ESM.docx]

**SUPPLEMENTAL INFORMATION**

**Table of Contents**

**Supplementary Table 1: Means and standard deviations of cytokines**

**measured via multiplex enzyme linked immunosorbent assay (ELISA) across**

**both conditions and all four time points………………………………………………………..2**

**Supplementary Table 2:** **Immune biomarker concentrations modeled over time,**

**using a dummy code system with baseline as the reference point…………………………… 3**

**Supplementary Table 3: Modeling Hamilton Depression Rating Scale (HDRS)**

**values on immune biomarker concentrations immediately post-intervention,**

**along with time and treatment…………………………………………………………………..3**

**Appendix I: Performance metrics for multiplex cytokine assay……………………………...4**

**Table S1: Means and standard deviations of plasma cytokine concentrations measured via multiplex enzyme-linked immunosorbent assay (ELISA) across both conditions (whole body hyperthermia vs. sham) and all four time points.**

|  | | Sham WBH | | WBH | |
| --- | --- | --- | --- | --- | --- |
| Cytokine | Time | Mean (pg/mL) | SD | Mean (pg/mL) | SD |
| IFNγ | B1 | 0.71 | 0.84 | 2.10 | 5.14 |
| IFNγ | B2 | 0.65 | 0.81 | 1.81 | 3.97 |
| IFNγ | B3 | 0.78 | 0.91 | 2.05 | 5.16 |
| IFNγ | B4 | 0.65 | 0.69 | 1.71 | 3.73 |
| IL-1β | B1 | 13.04 | 15.72 | 49.40 | 89.47 |
| IL-1β | B2 | 11.91 | 10.87 | 49.52 | 81.06 |
| IL-1β | B3 | 11.10 | 11.42 | 44.19 | 72.05 |
| IL-1β | B4 | 13.77 | 13.54 | 44.22 | 67.30 |
| IL-1ɑ | B1 | 1.88 | 2.70 | 7.92 | 23.52 |
| IL-1ɑ | B2 | 1.66 | 2.61 | 7.67 | 22.21 |
| IL-1ɑ | B3 | 1.26 | 2.00 | 6.96 | 19.10 |
| IL-1ɑ | B4 | 1.02 | 1.24 | 8.58 | 24.79 |
| IL-4 | B1 | 0.73 | 0.87 | 2.88 | 7.09 |
| IL-4 | B2 | 0.79 | 0.82 | 4.08 | 11.42 |
| IL-4 | B3 | 0.81 | 0.88 | 3.54 | 9.40 |
| IL-4 | B4 | 0.87 | 0.96 | 3.79 | 9.37 |
| IL-6 | B1 | 1.70 | 0.91 | 5.25 | 11.93 |
| IL-6 | B2 | 2.31 | 0.90 | 13.93 | 26.86 |
| IL-6 | B3 | 2.04 | 1.38 | 7.33 | 20.10 |
| IL-6 | B4 | 1.62 | 0.82 | 6.08 | 13.31 |
| IL-8 | B1 | 15.93 | 12.80 | 39.18 | 38.41 |
| IL-8 | B2 | 20.38 | 13.65 | 42.36 | 42.57 |
| IL-8 | B3 | 19.82 | 14.27 | 37.54 | 42.34 |
| IL-8 | B4 | 20.60 | 13.27 | 42.64 | 39.93 |
| IL-10 | B1 | 1.85 | 1.09 | 4.71 | 6.79 |
| IL-10 | B2 | 1.61 | 0.92 | 5.29 | 8.63 |
| IL-10 | B3 | 2.66 | 2.59 | 5.33 | 9.58 |
| IL-10 | B4 | 1.88 | 1.05 | 4.42 | 5.74 |
| IL-12p70 | B1 | 20.89 | 27.59 | 68.38 | 163.98 |
| IL-12p70 | B2 | 9.60 | 17.65 | 93.20 | 247.38 |
| IL-12p70 | B3 | 14.00 | 21.44 | 112.30 | 320.24 |
| IL-12p70 | B4 | 7.59 | 12.67 | 69.61 | 158.87 |
| TNF | B1 | 3.68 | 4.91 | 35.90 | 107.24 |
| TNF | B2 | 3.93 | 5.77 | 49.38 | 156.72 |
| TNF | B3 | 4.27 | 6.12 | 47.29 | 154.69 |
| TNF | B4 | 2.82 | 4.07 | 45.82 | 134.09 |

*Abbreviations: B1, baseline; B2, immediately post-intervention; B3, 1 week post-intervention; B4, 4 weeks post-intervention; IFN, interferon; IL, interleukin; TNF, tumor necrosis factor.*

**Table S2: Immune biomarker concentrations modeled over time, using a dummy code system with baseline as the reference point.**

| Line |  |  |
| --- | --- | --- |
| 1 | Metric_i_ = | β_0_ + |
| 2 | *(Time Effects)* | β_1_* DummyCode_1i_ + β_2_* DummyCode_2i_ + β_3_* DummyCode_3i_ + |
| 3 | *(Tx Effects)* | Β_4_*Tx_i_ + |
| 4 | *(Time*Tx)* | β_5_* DummyCode_1i_*Tx_i_ + β_6_*DummyCode_2i_*Tx_i_ + β_7_*DummyCode_3i_*Tx_i_ + ε_i_ |
|  | *Random Effects:* | |
| 5 | β_0_ = | γ_00_ + γ_01_*Participant_i_ + u_0_ |

*Abbreviations: Tx, treatment*

**Table S3: Modeling Hamilton Depression Rating Scale (HDRS) values on immune biomarker concentrations immediately post-intervention, along with time and treatment.**

| Line |  |  |
| --- | --- | --- |
| 1 | HDRS_i_ = | β_0_ + |
| 2 | *(Baseline Biomarker)* | β_1_*BaseBiomarker_i_ + |
| 3 | *(Time Effects)* | Β_2_* LogTime_i_ + |
| 4 | *(Treatment Effects)* | Β_3_*Tx_i_ + |
| 5 | *(Biomarker)* | Β_4_*Biomarker_i_ + |
| 6 | *(Time*Tx)* | Β_5_*LogTime_i_*Tx_i_ + |
| 7 | *(Time*Biomarker)* | Β_6_*LogTime_i_*Biomarker_i_ + |
| 8 | *(Time*Biomarker*Tx)* | Β_7_*LogTime_i_*Biomarker_i_*Tx_i_ + ε_i_ |
|  | *Random Effects:* | |
| 9 | β_0_ = | γ_00_ + γ_01_*Participant_i_ + u_0_ |

*Abbreviations: HDRS, Hamilton Depression Rating Scale; Tx, treatment*

|  |  | | **IFNγ** | **IL-1**α | **IL-1β** | **IL-4** | **IL-6** | **IL-8** | **IL-10** | **IL-12p70** | **TNF**α |
| --- | --- | --- | --- | --- | --- | --- | --- | --- | --- | --- | --- |
| **Average of plates 1-3**^1^ | **%CV** | | **12.2%** | **20.0%** | **12.3%** | **19.3%** | **6.3%** | **9.0%** | **6.8%** | **18.0%** | **15.5%** |
|  | Samples with %CV > 20 (total samples) | | 16 (119) | 33 (119) | 23 (119) | 32 (119) | 4  (119) | 8  (119) | 5 (119) | 32 (119) | 22 (119) |
|  | Samples < LLOQ (total samples) | | 7  (119) | 2  (119) | 1  (119) | 7  (119) | 0  (119) | 0  (119) | 0  (119) | 25  (119) | 28  (119) |
|  | Samples > ULOQ (total samples) | | 0  (119) | 0  (119) | 0  (119) | 0  (119) | 0  (119) | 0  (119) | 0  (119) | 0  (119) | 0  (119) |
| **Aushon %CV^2^** | C1  High Control | Mean (pg/mL) | 8.74 | 513.70 | 96.32 | 61.46 | 30.90 | 185.90 | 82.94 | 478.20 | 232.70 |
|  |  | %CV | 2.7% | 5.5% | 6.3% | 3.4% | 4.6% | 3.1% | 3.6% | 3.4% | 4.8% |
|  | C2 Medium Control | Mean (pg/mL) | 2.60 | 67.21 | 18.08 | 6.27 | 5.63 | 33.21 | 15.83 | 80.02 | 42.55 |
|  |  | %CV | 4.4% | 7.6% | 6.2% | 8.8% | 3.6% | 4.3% | 4.0% | 4.1% | 3.4% |
|  | C3  Low Control | Mean (pg/mL) | 0.28 | 7.51 | 0.68 | 0.47 | 0.34 | 1.62 | 0.56 | 4.41 | 8.88 |
|  |  | %CV | 6.8% | 4.5% | 5.3% | 3.3% | 6.4% | 5.9% | 5.5% | 4.4% | 4.6% |
|  | **Mean** | **%CV** | **4.6%** | **5.9%** | **5.9%** | **5.1%** | **4.9%** | **4.4%** | **4.5%** | **4.0%** | **4.3%** |

**Appendix I.** **Performance metrics for multiplex cytokine assay.**

**Table 1.** Intra-assay coefficients of variation.

^1^Table provides intra-assay percent coefficients of variation (%CV) for the multiplex assay used, calculated for each cytokine from samples run in duplicate across all three plates in the assay. Also shown are the number of samples that were below the lower limits of quantification (LLOQ) for each cytokine, relative to the total number of samples in the assay, and the number of samples that were above the upper limits of quantification (ULOQ) for each cytokine, relative to the total number of samples in the assay.

^2^Manufacturer-provided intra-assay coefficients of variation. Three levels of lyophilized controls in a biological matrix were reconstituted in DI water and diluted 1:2 in Sample Diluent. Each control level was assayed in 24 wells of the Aushon Human Cytokine Array Kit. The mean concentration (uncorrected for dilution) and % CV based on concentration are reported in the table above.

**Table 2.** Inter-assay coefficients of variation.

|  |  | | **IFNγ** | **IL-1**α | **IL-1β** | **IL-4** | **IL-6** | **IL-8** | **IL-10** | **IL-12p70** | **TNF**α |
| --- | --- | --- | --- | --- | --- | --- | --- | --- | --- | --- | --- |
| **%CV^1^** | Plate 1 (pg/mL) | | 1.1 | 33.9 | 0.5 | 3.3 | 1.5 | 28.8 | 4.0 | 1.22 | 0.69 |
|  | Plate 2 (pg/mL) | | 0.6 | 40.8 | 0.4 | 2.5 | 1.9 | 36.4 | 2.6 | 1.9 | 1.38 |
|  | Plate 3 (pg/mL) | | 1.2 | 39.9 | 0.6 | 3.5 | 1.7 | 29.36 | 4.0 | 1.0 | 0.5 |
|  | **%CV** | | **33.5%** | **9.8%** | **18.2%** | **17.4%** | **10.1%** | **13.4%** | **23.1%** | **33.8%** | **55.4%** |
| **Aushon %CV^2^** | C1  High | Mean (pg/mL) | 11.75 | 1014.09 | 185.95 | 138.99 | 58.74 | 385.94 | 142.33 | 948.24 | 471.31 |
|  |  | %CV | 9.0% | 10.2% | 4.1% | 10.9% | 4.1% | 2.5% | 3.9% | 2.8% | 4.7% |
|  | C2 Medium | Mean (pg/mL) | 3.40 | 130.09 | 34.89 | 17.22 | 11.49 | 80.36 | 26.85 | 168.31 | 72.72 |
|  |  | %CV | 6.5% | 12.3% | 6.7% | 6.5% | 3.8% | 8.0% | 2.2% | 3.3% | 4.8% |
|  | C3  Low | Mean (pg/mL) | 0.60 | 16.49 | 1.40 | 1.06 | 0.78 | 3.93 | 1.13 | 9.73 | 18.57 |
|  |  | % CV | 14.8% | 12.1% | 9.8% | 8.8% | 8.4% | 9.0% | 5.5% | 7.7% | 5.2% |
|  | **Mean** | **%CV** | **10.1%** | **11.5%** | **6.9%** | **8.7%** | **5.4%** | **6.5%** | **3.9%** | **4.6%** | **4.9%** |

^1^Table provides inter-assay percent coefficients of variation (%CV) for the multiplex assay used, calculated for each cytokine using the average concentration (pg/mL) of a single sample run in duplicate across all three plates in the assay. Sample concentrations fit within the range of the lowest control from the Human Cytokine Array Kit Validation Report.

**^2^** Manufacturer-provided inter-assay coefficients of variation. Three levels of lyophilized controls in a biological matrix were reconstituted in DI water, diluted 1:2 in Sample Diluent, and assayed in the Aushon Human Cytokine Array Kit. The testing was conducted over eight assays run on multiple days with multiple operators using a single lot of plates and biotinylated antibody reagent. The mean concentration (corrected for dilution) and percent CV based on concentration are reported in the table above.

**Table 3**. Limits of detection (LOD), lower limits of quantification (LLOQ) and upper limits of quantification (ULOQ) for each cytokine.^1^

|  | **IFNγ** | **IL-1**α | **IL-1β** | **IL-4** | **IL-6** | **IL-8** | **IL-10** | **IL-12p70** | **TNF**α |
| --- | --- | --- | --- | --- | --- | --- | --- | --- | --- |
| **LOD**  **(pg/mL)** | 0.0115 | 0.210 | 0.0229 | 0.0145 | 0.0082 | 0.0853 | 0.0297 | 0.0663 | 0.0829 |
| **LLOQ**  **(pg/mL)** | 0.0122 | 0.4395 | 0.0488 | 0.0488 | 0.0278 | 0.3906 | 0.0488 | 0.2930 | 0.0977 |
| **ULOQ**  **(pg/mL)** | 50.0 | 1800 | 200 | 200 | 114 | 400 | 200 | 1200 | 400 |

^1^Table provides the manufacturer-provided limits of detection (LOD), lower limits of quantification (LLOQ), and upper limits of quantification (ULOQ) for each cytokine. The calculated LOD is the mean of zero calibration standards + 2 standard deviations; the LLOQ is the lowest calibration standard with back-calculated concentration CV <20% and relative error <25%; and the ULOQ is the highest calibration standard with back-calculated concentration CV <20% and relative error <20%. For all analytes, the LOD is below the low standard.
